# Immune Predictors of Response after Bacillus *Calmette–Guérin* Treatment in Non-Muscle-Invasive Bladder Cancer

**DOI:** 10.3390/cancers15235554

**Published:** 2023-11-23

**Authors:** Marta Rodríguez-Izquierdo, Carmen G. Del Cañizo, Carolina Rubio, Ignacio A. Reina, Mario Hernández Arroyo, Alfredo Rodríguez Antolín, Marta Dueñas Porto, Félix Guerrero-Ramos

**Affiliations:** 1 Department of Urology, University Hospital 12 Octubre, 28041 Madrid, Spain; carmen.gcanizo@gmail.com (C.G.D.C.); marioha13@gmail.com (M.H.A.); felixguerrero@gmail.com (F.G.-R.); 2 Molecular and Traslational Oncology Division, Biomedical Innovation Unit, CIEMAT, 28040 Madrid, Spain; carolina.rubio@externos.ciemat.es (C.R.); ignacio.arevalo@externos.ciemat.es (I.A.R.); marta.duenas@ciemat.es (M.D.P.); 3 Centro de Investigación Biomédica en Red Cáncer, 28029 Madrid, Spain; 4 Institute of Biomedical Research, University Hospital 12 de Octubre, 28041 Madrid, Spain; 5 Department of Urology, Sanchinarro Hospital (HM), 28050 Madrid, Spain

**Keywords:** bladder cancer, Bacillus *Calmette–Guérin*, cytokines, tumor microenvironment, immune response

## Abstract

**Simple Summary:**

Bladder cancer is the tenth most frequently diagnosed malignant disease globally. In total, 75% of these patients present with a non-muscle-invasive disease, a fraction of which will progress to muscle-invasive bladder cancer. Bacillus *Calmette–Guérin* is the standard of care treatment for high-risk, non-muscle-invasive bladder cancer (NMIBC), but predictors of response are lacking. There is a need to find more precise ways to identify non-responder patients in order to offer the most suitable treatment option.

**Abstract:**

Bacillus Calmette–Guérin (BCG) has been the standard of care for the treatment of high-risk, non-muscle-invasive bladder cancer (NMIBC) for decades, but 49.6% of high-risk and very-high-risk patients will experience progression to muscle-invasive disease in five years. Furthermore, cytology and cystoscopy entail a high burden for both patients and health care systems due to the need for very long periods of follow-up. Subsequent adjuvant treatment using intravesical immunotherapy with BCG has been shown to be effective in reducing tumor recurrence and progression, but it is not free of severe adverse effects that ultimately diminish patients’ quality of life. Because not all patients benefit from BCG treatment, it is of paramount importance to be able to identify responders and non-responders to BCG as soon as possible in order to offer the best available treatment and prevent unnecessary adverse events. The tumor microenvironment (TME), local immune response, and systemic immune response (both adaptive and innate) seem to play an important role in defining responders, although the way they interact remains unclear. A shift towards a proinflammatory immune response in TME is thought to be related to BCG effectiveness. The aim of this review is to collect the most relevant data available regarding BCG’s mechanism of action, its role in modulating innate and adaptive immune responses and the secretion of certain cytokines, and their potential use as immunological markers of response; the aim is also to identify promising lines of investigation.

## 1. Introduction

Bladder cancer (BC) is the tenth most common cancer worldwide [1] and the sixth in the developed world [2]. The disease is four times more prevalent in men than women. Tobacco smoking is the main risk factor associated with bladder cancer development, and it accounts for approximately 50–60% of cases [2,3], but other occupational and environmental toxins, such as aromatic amines, polycyclic aromatic hydrocarbons, and chlorinated hydrocarbons, have been correlated with urothelial cancer.

At diagnosis, about 75% of patients present with non-muscle-invasive bladder cancer (NMIBC), but this entity includes a very heterogeneous group. *Bacillus Calmette–Guérin* (BCG) is the standard of care adjuvant treatment for intermediate and high-risk disease. However, there is a 9.6% and a 40% probability of progression to muscle-invasive bladder cancer (MIBC) for high-risk and very-high-risk patients, respectively, so it is of paramount importance to identify as early as possible BCG-non-responsive patients. Furthermore, BCG is not free of adverse effects, and it is not always well tolerated. In recent years, a scarcity of BCG production has led to a BCG shortage, which has forced clinicians to be more selective with patients that receive the treatment. However, this selection is solely based on clinicopathological data, which, nowadays, are insufficient to predict response. For decades, the follow-up of patients with bladder cancer consisted of urine cytology and cystoscopy, but these two classic tests entail a high burden for both patients and health care systems and do not predict response to treatment. Therefore, there is a need to find predictive methods for the identification of non-responder patients [4]. 

Currently, there are several scoring models to try to identify patients at high risk of recurrence, such as the EORTC (European Organization for Research and Treatment of Cancer) and CUETO (Club Urológico Español de Tratamiento Oncológico) scores. More recently, the EAU NMIBC 2021 (European Association of Urology NMIBC 2021) scoring model has been described, which is the only one that uses both WHO 2004/2016 (World Health Organization) and WHO 1973 classification systems [5]. These are clinicopathological scores useful in clinical practice, but they do not consider individual patient response to BCG.

Fluorescence In Situ Hybridization (FISH) has been shown to be helpful in predicting recurrence after BCG treatment [6,7]. The FISH test is considered positive if four or more cells show polysomy on at least two chromosomes (3, 7, or 17) and/or at least twelve cells show a homozygous deletion for 9p21 [7]. In Liem et al.’s metanalysis [6], the FISH test is carried out at four time points: t0 (before transurethral resection of the bladder tumor, TURBT); t1 (after 6-week BCG induction); t2 (3 months after TURBT); and t3 (6 months after TURBT). A positive FISH test at either t1, t2, or t3 correlates with a higher risk of tumor recurrence (all *p* < 0.005), with an HR of 2.23, 3.70, and 23.44, respectively. The hazard ratio (HR) at t3 needs to be interpreted with caution due to the wide 95% CI range (5.25–104.49). FISH test results at t0 are not correlated with a higher risk of recurrence. In the trial developed by Kamat et al. [5], 57% of patients with a positive FISH result at 3 months developed recurrence at 24 months, which correlates with a 2-year recurrence-free survival of 43% and a 2-year progression-free survival of 71%. After multivariate analysis, only a positive FISH test 3 months after the initial TURBT correlated with a higher risk of both recurrence and progression. On the other hand, only 8% of patients with negative FISH results at 3 months after TURBT developed recurrence, and 3% developed progression.

More recent research has focused on the immune response after BCG treatment. Despite the best efforts of the scientific community, the BCG-induced response in bladder cancer is not completely understood. There seems to be a local immune response that induces a cascade of events that modifies the tumor microenvironment (TME), thus leading to systemic immune modulation. The combination of both local and systemic immune responses could be the key to understanding interpersonal variability regarding the response to BCG treatment. In this review, we aim to gather the most relevant published data to try to understand the mechanism of action of BCG and the immune response induced with this therapy. It is necessary to identify as early as possible, in a predictive manner and with high accuracy, patients at risk of recurrence or progression after or even during BCG treatment.

## 2. Methods 

We conducted a non-systematic review of the National Institutes of Health (PubMed) for articles published up to September 2023. The key words searched were “bladder cancer”, “BCG”, “cytokines”, “tumor microenvironment”, and “immune response.”

## 3. Mechanism of Action of BCG 

The TME, and, specifically, the immune-adaptive and innate component, are crucial for cancer-specific survival. One of the most important factors that determines BCG effectiveness is the patient’s ability to generate an appropriate immune response. There are different ways in which BCG modulates the immune system: by causing a direct cytotoxic effect and by activating innate and adaptive immune responses.

**Figure 1:**
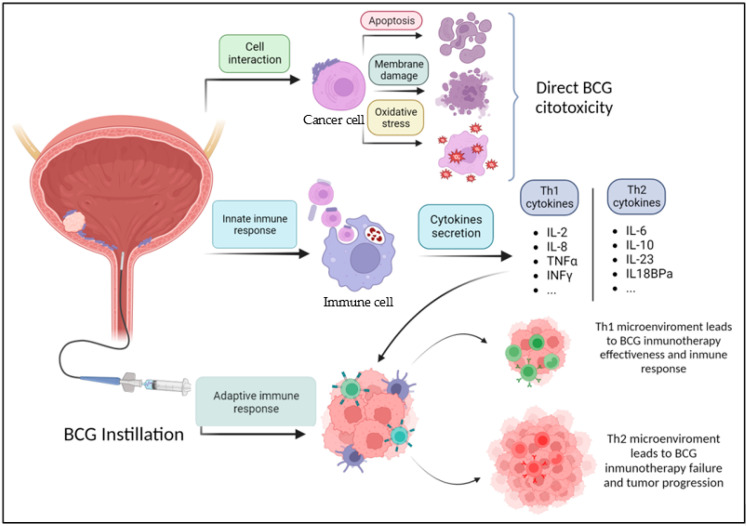
Mechanism of action of BCG. BCG: Bacillus *Calmette–Guérin.* Figure made in https://www.biorender.com/ (accessed on 27 October 2023).

It has been shown that BCG can directly cause damage to tumor cells, thus inducing apoptosis, necrosis, oxidative stress, and others [8] (Figure 1). Jiansong et al. demonstrated that BCG induces apoptosis by activating the caspase 8 signaling cascade, which eventually leads to apoptosis after the activation of the toll-like receptor 7 (TLR7). BCG can also cause cell necrosis through cell membrane integrity damage through the release of necrosis associated chemokine high molecular group box protein 1 (HMGB1) [9]. Another mechanism through which BCG induces cellular damage is by generating intracellular oxidative stress. Bladder cancer cells internalize BCG, which increases the production of nitric oxide (NO) through inducible nitric oxide synthase (iNOS) [8]. High levels of NO are known to have a cytotoxic effect on urothelial cancer cells [10,11]

### 3.1. Immunological Markers

#### 3.1.1. Immune Cells from the TME

BCG induces cellular changes that activate both innate and adaptive immune responses. Regarding the innate immune response, both tumor cells and innate immune response cells secrete cytokines after BCG stimulation. Neutrophils can kill tumor cells directly through phagocytosis, and they secrete tumor necrosis factor-related apoptotic ligand (TRAIL), which is a member of the TNF family that induces apoptosis in bladder cancer cells [12].

It has also been demonstrated that macrophage polarization in TME plays an important role in the tumor microenvironment, and it has been correlated with tumor progression [13]. The role of macrophages in BC differs depending on their phenotype and localization [14]. M2-like polarization of tumor associated macrophages (TAMs) is associated with higher tumor grade, poor response to BCG, and worse prognosis [15]. There is an increase in TAMs that correlates with tumor progression, which suggests a role of these cells in aggressiveness and poorer clinical outcomes [14]. TAMs have also been correlated with tumor recurrence in NMIBC [16]. However, M1-like macrophages in tumor draining lymph nodes [17] have been correlated with positive outcomes. Macrophages display a dual role: they serve as an APC and, once activated, they can, in a non-specific way, phagocytize tumor cells. In addition, macrophages can secrete macrophage secretory factors (MSFs) that induce NO production. Regarding macrophage response after BCG exposure, there is also controversy. On the one hand, several in vitro experiments have proved that BCG treatment induces the production of Th1- cytokines in macrophages, and that it favors a macrophage-mediated cytotoxicity against bladder cancer cells [18,19,20]. Macrophages are believed to be recruited to the bladder wall from monocytes through chemokine production [14]. These chemokines can be secreted by both normal and cancerous urothelial cells, as well as PBMCs following BCG administration [21]. On the other hand, BCG can also induce pro-tumor functions in macrophages, such as an increase in macrophage-secreted IL10, that reduce the cytotoxic activity of macrophages themselves [22]. It has also been demonstrated that BCG-stimulated macrophages support the proliferation and activation of fibroblasts, which can favor tumor progression [23] A hypothesis for this opposite role is that the beneficial effect of macrophages comes from freshly recruited macrophages after BCG administration, not from the macrophages already in the TME (which are M-2, like TAMS, and actually correlate with poorer outcomes).

Among the effector cells of the innate immune response, NK cells have their own prominent role in BCG-induced cytotoxicity. Even when NK cells are not a major immune cell population in the bladder wall, Brandau et al. [24] demonstrated their fundamental role for BCG antitumor response using a syngeneic mouse model depleted of NK cells through the use of anti-NK1.1 monoclonal antibody. They demonstrated the BCG-induced cytotoxicity of human NK cells in vitro and the failure of BCG immunotherapy in mice lacking NK cell activity.

In relation to the adaptive immune response, BCG attaches to tumor cells through the interaction between fibronectin on the surface of tumor cells and fibronectin attachment protein (FAP) on the BCG wall surface [25]. After the internalization of BCG or phagocytosis by macrophages, antigen-presenting cells (APC) process BCG and present its antigens to CD4+ T cells and CD8+ T cells [8]. This immune response generates a cascade of events that results in a secretion of different cytokines (T helper type 1 –Th1- cytokines and T helper type 2 –Th2-cytokines).

Nunez-Nateras et al. [26] studied the TME of patients with Tis. BCG is known to act as a localized Th1-polarizing immune modulator. They observed that patients with a pre-BCG tumor microenvironment that was already polarized to Th1 (T-bet^+^) did not respond to BCG therapy. On the other hand, patients with a pre-BCG tumor microenvironment polarized to Th-2 (GATA-3+) were responders to BCG due to the shift in the immune polarization that activated a massive influx of inflammatory cells. Patients whose tumor microenvironment has already escaped to the Th-1 response are less likely to respond to any therapy that polarizes the inflammatory response to Th-1. In that study, they found that a significantly lower level of T-bet+ cells was identified among BCG responders, without any significant change in GATA-3+ cells. This reinforces the hypothesis that it is the lower peritumoral infiltration of Th1 cells that makes the difference in the immune response, regardless of the Th2 cell count in the peritumoral tissue. 

#### 3.1.2. Systemic Immune Cells

PD-L1 (programmed cell death-ligand) is an immune checkpoint expressed in tumor cells, and it binds to its receptor (PD-1), which is expressed on the surface of T cells. This binding enables the tumor cell to escape the immune system. This discovery revolutionized the scientific community, and it is the rationale for the development of various immune checkpoint inhibitors approved since then [27]. PD-L1 is expressed by high-grade urothelial carcinoma, and it is correlated with tumor recurrence and poorer survival [28]. It has been associated with resistance to BCG therapy [29]. BCG is known to recruit CD8+ T cells into the TME and secrete INFγ as an initiation to the cytotoxic immune response. These pathways, however, upregulate PD-L1 in cancer cells to evade immune recognition [30]. This is known as adaptive immune resistance, and it can be one of the mechanisms of BCG failure in up to 25% of patients [31], which is why there are currently ongoing clinical trials combining BCG and immune checkpoint inhibitors [32,33,34]. Some in vivo and in vitro trials have demonstrated that the number and activity of tumor-infiltrating CD8+ Tcells increase significantly when BCG and anti-PD-L1 drugs are combined. However, the relationship between the expression of PD-L1 in tumor cells and the TME in NMIBC patients and the response rate to BCG treatment is unclear. In some trials, BCG has been shown to downregulate the expression of PD-L1 in bladder cancer cells [35].

#### 3.1.3. Cytokines

The activity and efficacy of BCG are, in great part, due to the immune response mediated by cytokines. There is a large amount of evidence of the presence of cytokines in urine and serum post-BCG instillation, including IL1, IL2, IL6, IL8, IL10, IL12, TNFα, and INFγ [36] (Table 1).

The main cytokines that mediate the Th1 immune response include INFγ, IL2, IL12, and TNFα, among others. Some of these cytokines appear right after the first instillation, such as IL1, IL6, IL8, and IL12, and some others appear after a few instillations. This can reflect the origin of these cytokines: local macrophages secrete IL1 and IL6, but other cytokines, such as IL2 and INFγ, are produced only after T-cell activation that occurs after repeated BCG instillations [37].

Kamat et al. [38] proposed a panel of nine urinary cytokines (CyPRIT) that predicted the likelihood of recurrence in patients with intermediate and high-risk non-muscle-invasive bladder cancer after BCG treatment with 85.5% accuracy (95% CI, 77.9–93.1%). Although what they proposed is a self-developed nomogram, some results, such as the correlation of IL6 and IL8 with recurrence, are in agreement with previous publications. In a phase II study, Salmasi et al. [39] studied a panel of 105 urinary cytokines at various points during treatment with BCG in intermediate and high-risk NMIBC with and without the administration of the HS-410 vaccine. These patients received BCG induction for 6 weeks followed by a maintenance consisting of a weekly treatment of BCG for 3 weeks at months 3, 6, and 12. Urine cytokines were measured at different points after treatment (prior to BCG and at weeks 7, 13, and 28) to predict treatment failure in intermediate and high-risk NMIBC patients. There was no significant difference in baseline cytokine levels in responders versus non-responders. At week 13, the increased percent change of IL18BPa and IL23 and the decreased percent change of IL8 and IP10 (INFγ induced protein 10, also known as CXCL10) from baseline were predictors of treatment failure. Furthermore, lower levels of ITAC (INF-inducible T-Cell α chemoattractant), IL1b, IL2, IL16, and macrophage inflammatory protein (MIP-1a/MIP1-b) were predictors of a higher rate of recurrence. IP10 is known to act as a chemoattractant for regulatory T-cells. It has been reported that both an increase and a decrease in urinary levels were associated with poor recurrence-free survival [40,41], so there is a need to clarify their role in bladder cancer patients. ITAC plays an important role in inducing Th1-type immune response. Ultimately, urinary levels of IP10, resisting (an adipokine secreted from monocytes and macrophages), and SHGB were associated with time-to-treatment failure. These markers are, in some way, related to the Th1-type immune response by either inducing it (e.g., IPAC, IL8, IL2, and IL16) or inhibiting it (IL18BPa and IL23), which correlates with longer or shorter failure-free survival, respectively. 

Ashiru et al. [42] screened a panel of cytokines and chemokines in urine one week after BCG instillations in a cohort of 12 patients with NMIBC. They compared these cytokines with a control of six patients who received intravesical mitomycin C (MMC). MMC was considered a good control because it does not cause a significant amount of inflammation or activation of the immune response due to its different anti-tumor mechanism of action. Among the wide range of cytokines tested, IP10 showed an increased pattern in all responders after BCG treatment. Two of the twelve patients presented BCG toxicity. These patients had very high levels of urinary IP10 (above 800 pg/mL), suggesting that an exaggerated amount of this urinary chemokine may correlate with an excessive inflammatory response that provokes patient discomfort. In addition, the levels of IL6 and IL8 were also unusually elevated in patients who experienced adverse effects. In contrast, the two patients in this cohort who recurred (during a period of 5 years) had very low IP10 levels, if any. This chemokine is produced by myeloid cells. To better understand the role of IP10, in vitro incubation of peripheral mononuclear blood cells (PBMC) with high concentrations of this chemokine was performed. They observed that the main role of IP10 is attracting effector cells (including T CD3 and anti-tumor CD56 bright NK cells) towards the bladder in BCG-treated patients.

In a more recent study by Elsawy et al. [43], different urinary cytokines were measured at different time points before and after a BCG 6-week induction in 204 patients with high-risk bladder cancer (T1HG with or without concomitant CIS). In that study, urinary levels of both IL2 and TNFα (Th1 cytokines) increased after BCG instillation in all patients, regardless of responsiveness. Serum TNFα mean fold change was a significant predictor of initial complete response (with no evidence of tumor upon cystoscopy at 3 months), but no significant role was demonstrated in relation to recurrence or progression. The change pattern of IL10 urinary levels (Th2 cytokine) was the only immunological predictor of BGC unresponsiveness. Increased urinary levels of IL10 after BCG were significantly associated with recurrence and progression. Other immunological markers, such as CTLA-4 (an immunomodulatory mediator that downregulates T-cell activation, leading to the suppression of the anti-tumor BCG response), were also evaluated, and decreased levels of serum CTLA-4 were associated with a good response to treatment. In concordance with previous studies, they also found, in this cohort, that a higher GATA3+/Tbet+ ratio after BCG induction was significantly associated with tumor recurrence and progression. As mentioned above, T-bet+ is known to regulate Th1 differentiation. 

Urinary levels of IL1, IL2, IL6, IL10, IL8, TNFα, and TRAIL have been evaluated as predictors for treatment response after BCG. Elevated IL8 or IL18 expression in the first hours after BCG treatment is associated with longer disease-free survival in patients with NMIBC [37]. However, there is controversy regarding the IL8 urinary level and the response to BCG. Kai et al. [44] found that elevated levels of IL8 in urinary samples were significantly associated with tumor recurrence after BCG treatment. This difference may be explained by the differences in time when IL8 was measured. In Jackson et al.’s study [37], IL8 was measured in urine samples in the first hours after BCG administration, while in Kai et al.’s trial [44], the measurement was made in urine a mean of 160 days after BCG instillation. Urinary TRAIL levels appear to be increased in BCG-responsive patients compared with non-responders [45].

**Table 1:** Classification of cytokines and their relationship with immune response after BCG instillations. FFS (failure-free survival); DFS (disease-free survival); BCG (Bacillus Calmette–Guérin); ICR (initial complete response; in Elsawy et al.’s trial, this is defined as tumor-free 3 months after biopsy).

	Cytokine	Th Type	Found In	Cell That Produces	Tumor Effect After BCG Instillation	Reference
Anti-tumoral effect	IL2	Th1	Urine, tissue	T-cell	Related to longer FFS; lower levels related to higher rate of recurrence	Xiaoxuan Liu et al. [36], Jackson AM et al. [37], Salmasi et al. [39], Videira et al. [40]
	IL8	Th1	Urine	Monocytes/macrophages/dendritic cells	Higher levels immediately after BCG are related to longer FFS, longer DFS	Kamat et al. [38]
	IL18	Th1	Urine	Monocytes/macrophages	Higher levels after BCG are related to longer FFS	Kamat et al. [38]
	TNFα	Th1	Serum	T-cell	Increased in all patients after BCG. Mean fold change was predictor of ICR	Elsawy et al. [43]
	INFγ	Th1	Urine	T-cell	Higher levels after BCG are related to longer FFS	Kamat et al. [38]
	IP10 (CXCL10)	Th1	Tissue, urine	Myeloid cells	Chemoattractant for regulatory T-cells; increase in BCG responders; attracts effector cells	Videira et al. [40], Ashiru et al. [42]
	ITAC	Th1	Urine	Leucocytes	Lower levels after BCG are related to higher rate of recurrence	Kamat et al. [38]
	IL16	Th1	Urine	Monocytes/macrophages	Lower levels related to higher rate of recurrence	Kamat et al. [38]
	MIP-1a, MIP1-b	Th1	Tissue	Leucocytes	Elevated in tissue samples in BCG-responder patients	Videira et al. [40]
	TRAIL	-	Urine	Leucocytes	Increased in BCG-responder patients	Kamat et al. [38]
Pro-tumoral effect	IL6	Th2	Urine	Local macrophages	Higher levels associated with recurrence and progression	Jackson AM et al. [37], Kamat et al. [38]
	IL10	Th2	Urine	CD4+ cells	Lower levels of urinary IL10 were correlated with lower rate of progression after BCG treatment	Elsawy et al. [43]
	IL18BPa	Th 2	Urine	Leucocytes	Increased percent change after BCG was correlated with shorter FFS, treatment failure	Salmasi et al. [39], Kamat et al. [38]
	IL23	Th2	Urine	Macrophages and dendritic cells	Increased percent change after BCG was correlated with shorter FFS, treatment failure	Salmasi et al. [39], Kamat et al. [38]
	CTLA-4	Th2	Serum	T-cells	Lower levels of serum CTLA-4 were correlated with a lower rate of progression	Elsawy et al. [43]
	SHGB	-	Urine		Higher urinary levels at week 13 post BCG correlated with worse FFS	Salmasi et al. [39]

Wenlong Zhong et al. [46] evaluated several serum cytokines to assess the systemic BCG immune response to identify potential responders. They selected CCL27, the cytokine with the most predictive value within those studied in their trial. CCL27 (also known as T-cell attracting chemokine) has been found to be involved in tumor progression, metastasis, and immune escape in other types of cancers [47,48]. Among their cohort of 37 patients, 28 were responders, and 9 were classified as BCG unresponsive, with recurrence within the first 6 months after initiating BCG therapy. Serum CCL27 levels prior to treatment were significantly higher in the non-responders group compared with responders, with an AUC of 0.73. When studying the TME, the infiltration of most immune cell types (macrophages, CD4T+ cells, and CD8T+ cells) did not correlate with the serum levels of CCL27. However, CCL27 had a strong positive correlation with the density of Treg (a subpopulation of T cells that modulates the immune response) in the TME in their cohort of high-risk NMIBC patients. Increased levels of Treg cells in the TME was significantly correlated with a poor recurrence-free survival after BCG. CCL27 changed heterogeneously after BCG instillation. It was observed that non-responders had a higher change rate of CCL27 at all time points during induction therapy. An increase in serum CCL27 over 89.16 pg/ml from baseline to the last time point (after 6-week induction therapy) was the best predictor of the BCG response, with 89% sensitivity and 68% specificity. After calculating a combined score of baseline CCL27 and the dynamic changes of CCL27 during treatment, the AUC of the combined score was 0.897 (95% CI 0.790–1.000, *p* < 0.001). 

## 4. New Strategies to Improve BCG Efficacy 

Due to the lack of effective treatments to replace BCG instillations, there is a need for improved BCG efficacy. Some researchers have tried to develop new strategies to address this issue. N-803 (ALT-803), which is an IL15 cytokine antibody fusion protein, is a complex of the IL15 superagonist and the dimer IL15 receptor α Su/IgG1 Fc fusion protein, and it has been studied in combination with other agents in preclinical trials, which revealed an additive tumor suppression effect [49,50,51]. IL15 ultimately increases the proliferation and activation of NK cells and CD8+ T cells. N-803 contains a N72D mutation in the IL15 sequence to increase the biological activity of IL15. Rosser et al. [52] developed a phase Ib trial combining BCG treatment with intravesical instillation of N-803 in increasing doses (up to 400 mcg per instillation) in high-risk NMIBC patients. Small changes in urinary cytokines were observed (IL2, IL10, TNFα, and INFγ). The urinary level of IL6 presented a more significant increase compared to baseline. The serum levels of IL2, IL10, IL4, TNFα, and INFγ remained unchanged. After a mean follow-up of 65.2 months, all patients were disease free, none had progressed, and only one experienced recurrence. Historical data would suggest a recurrence of at least 30%, with 10–20% progression among the recurrent patients. The most frequent adverse effects were hypertension (present in two thirds of patients), haematuria, fatigue, and urinary frequency. Most adverse effects were grade 1 or 2, but five patients presented with grade 3 hypertension (not considered treatment related) and one presented with grade 3 haematuria. 

A larger study was carried out with N-803 in BCG-unresponsive patients by Chamie et al. [53]. In this trial, there were two cohorts being studied: cohort A (n = 83; included persistent or recurrent CIS +/− recurrent Ta/T1) and cohort B (n = 77; included recurrent high-grade Ta/T1). Both cohorts received intravesical N-803 (400 microg) and BCG (standard dose) for 6-week induction and maintenance up to 3 years. In total, 50% of patients in cohort A presented a complete response at 6 months. The duration of response at 12 and 18 months was 37% and 24%, respectively. N-803 was well tolerated, with grade 1–2 dysuria, pollakiuria, and haematuria as the most frequent adverse events (22%, 19%, and 10% of patients, respectively). No grade 4 adverse events were described, and less than 1% were grade 3. More studies are needed to confirm these results and to evaluate this novel molecule in the BCG-naive setting. The FDA received (23 May 2022) and accepted to review (28 July 2022) the marketing submission of N-803 plus BCG for the treatment of BCGu NMIBC.

## 5. Systemic Immune Response

Not only is the TME important to predict response, but systemic immunological parameters have also been correlated with bladder cancer outcome. There are different clinical indicators in the peripheral blood that have been studied, with promising results.

Lim et al. [54] hypothesized that changes in the TME produced by BCG could be reflected in the peripheral blood. In a discovery cohort of five patients and a validation cohort of twenty-eight patients, they took samples of peripheral blood at three time points: prior to TURBT, after three instillations of BCG, and after the completion of BCG induction. Tissue samples were obtained during TURBT (both tumoral and normal mucosa samples) and after 6-week induction BCG to confirm the absence of neoplastic urothelial cells. In that study, NK and T cells (CD4+, CD8+, and Tregs) were decreased in the peripheral blood 3 months after BCG treatment, suggesting a recruiting of immune cells after BCG administration. This same group of researchers further studied TME by analyzing four main T cells present in the TME of 29 NMIBC patients (21 responders and 8 non-responders). The four major T cell subsets were: CD4+FOXP3+ Treg, CD4+FOXP3- non-Treg, CD8+PD-1+, and CD8+PD-1- T cells. They found higher baseline (pre-BCG) densities of CD4+FOXP3- non-Treg cells (*p* = 0.024) and CD8+PD-1+ T cells (*p* = 0.001) in responders versus non-responders, and this correlated with a longer RFS. However, 3 months after BCG, a higher rate of non-Treg CD4+FOXP3- and active CD8+PD-1- was found in the TME of responder patients (*p* = 0.0098 and *p* = 0.009, respectively). A higher density of CD8+PD-1+ was found (*p* = 0.03). This last finding supports the hypothesis of combining anti-PD-1 treatment in NMIBC patients to potentially increase the number of responder patients by preventing resistance to BCG.

A high neutrophil and/or monocyte count and a low absolute lymphocyte count correlate with poor prognosis in various types of cancer [55]. Furthermore, the neutrophil to lymphocyte ratio (NLR) has been associated with poor clinical outcomes in patients with muscle-invasive bladder cancer (MIBC), and it seems to correlate with progression and recurrence in non-muscle-invasive bladder cancer patients [56]. There are other systemic indicators that have been studied, such as the platelet-to-lymphocyte ratio (PLR), the monocyte-to-lymphocyte ratio (MLR), and the systemic inflammatory response index (SIRI), which includes neutrophils, monocytes, and lymphocytes (SIRI = neutrophil count x monocyte count/lymphocyte count). SIRI includes the count of three different immune cell types, and a high SIRI has been reported to correlate with shorter median RFS (recurrence-free survival) and PFS (progression-free survival) in NMIBC patients after BCG treatment [57]. In a retrospective study, blood samples of patients prior to surgery were analyzed to calculate the different inflammatory indices (PLR, NLR, MLR, and SIRI). A high SIRI value was positively associated with BCG non-response. These results are in line with other studies that reported that SIRI correlates with recurrence and progression in other malignant tumors (e.g., esophagus, hepatocarcinoma, and pancreatic cancer) [58,59,60].

In preclinical models, rapamycin enhances BCG vaccine efficacy and the killing capacity of *γ*δ T cells [61], which are an important component of the innate immune response [62]. Niannian Ji et al. [63] hypothesized that given that rapamycin (as an mTOR inhibitor) improves antigen-specific immunity, it could facilitate T cell response, which is essential for BCG efficacy. That study met its primary endpoint by demonstrating a statistically significant increased number of *γ*δ T cells in the peripheral blood and improving NK cell activation in patients receiving 2 mg of oral rapamycin. Adverse events were not greater than expected, and they were similar in both the treatment and placebo group. More studies are needed to assess if this improvement in innate immune response correlates with an improved clinical efficacy of the combination. 

There are not many studies combining both the intratumoral and systemic immune responses to evaluate the response to BCG treatment. Martínez et al. [64] observed that BCG responders show a lower level of peritumoral Th1 in comparison to non-responders, with no clear difference in Th2 count among responders and non-responders. Furthermore, a higher NLR value prior to BCG was observed among non-responders, although the difference did not reach statistical significance. Nonetheless, when a new immune score GTR/NRL was calculated (GTR = Ratio Th2/Th1) combining local and systemic inflammation values, BCG responders had a significantly higher ratio than BCG non-responders (*p* = 0.004). These data show that although we have not been able to find the relationship yet, there is probably some kind of correlation between the intratumoral and systemic immune responses. 

## 6. Conclusions

The immune response after BCG treatment is complex and not fully understood. A local and systemic immune mechanism seems to be responsible for the effect of BCG. There appears to be a consensus that a shift towards a proinflammatory immune response polarization (Th1) on the TME after BCG administration is essential for BCG effectiveness.

Cytokines are secreted by different cells, and they perform a number of different activities, which makes it difficult to classify them in this setting. Current knowledge in this area comes from small, single-center studies, which limits the extrapolation of their results. However, results from trials combining BCG and immune checkpoint inhibitors are still pending, which could change the standard of care treatment in these patients. Promising lines of investigation, such as the measurement of urinary cytokines, like CCL27, Cyprit nomogram, or systemic immune indicators (SIRI), could be useful for identifying BCG-unresponsive patients, which could lead to better selection of treatment, avoid unnecessary adverse events, and prevent a delay in radical cystectomy when necessary. Novel strategies to improve the efficacy of BGC in BCGu patients, like the intravesical administration of N-803, are pending FDA approval.

## 7. Limitations

The main limitations of this review are its non-systematic approach and the heterogeneity of the studies, which use very different methodologies, so the conclusions drawn have to be taken with caution.
